# Arginine regulates HSPA5/BiP translation through ribosome pausing in triple-negative breast cancer cells

**DOI:** 10.1038/s41416-023-02322-x

**Published:** 2023-06-29

**Authors:** Christina M. Vidal, Ching Ouyang, Yue Qi, Carlos Mendez-Dorantes, Alaysia Coblentz, Jackelyn A. Alva-Ornelas, Jeremy M. Stark, Victoria L. Seewaldt, David K. Ann

**Affiliations:** 1 Department of Diabetes Complications and Metabolism, Arthur Riggs Diabetes & Metabolism Research Institute, Beckman Research Institute of City of Hope, Duarte, CA 91010 USA; 2 Irell & Manella Graduate School of Biological Sciences, Beckman Research Institute of City of Hope, Duarte, CA 91010 USA; 3 Department of Computational and Quantitative Medicine, Beckman Research Institute of City of Hope, Duarte, CA 91010 USA; 4 Department of Cancer Genetics and Epigenetics, Beckman Research Institute of City of Hope, Duarte, CA 91010 USA; 5 Department of Population Sciences, Beckman Research Institute of City of Hope, Duarte, CA 91010 USA

**Keywords:** Cancer metabolism, Translation

## Abstract

**Background:**

Triple-negative breast cancer (TNBC) is an aggressive subtype of breast cancer with a high mortality rate due to a lack of therapeutic targets. Many TNBC cells are reliant on extracellular arginine for survival and express high levels of binding immunoglobin protein (BiP), a marker of metastasis and endoplasmic reticulum (ER) stress response.

**Methods:**

In this study, the effect of arginine shortage on BiP expression in the TNBC cell line MDA-MB-231 was evaluated. Two stable cell lines were generated in MDA-MB-231 cells: the first expressed wild-type BiP, and the second expressed a mutated BiP free of the two arginine pause-site codons, CCU and CGU, termed G-BiP.

**Results:**

The results showed that arginine shortage induced a non-canonical ER stress response by inhibiting BiP translation via ribosome pausing. Overexpression of G-BiP in MDA-MB-231 cells promoted cell resistance to arginine shortage compared to cells overexpressing wild-type BiP. Additionally, limiting arginine led to decreased levels of the spliced XBP1 in the G-BiP overexpressing cells, potentially contributing to their improved survival compared to the parental WT BiP overexpressing cells.

**Conclusion:**

In conclusion, these findings suggest that the downregulation of BiP disrupts proteostasis during arginine shortage-induced non-canonical ER stress and plays a key role in cell growth inhibition, indicating BiP as a target of codon-specific ribosome pausing upon arginine shortage.

## Background

Triple-negative breast cancer (TNBC) is a highly heterogeneous, aggressive breast cancer subtype with a poor prognosis for which no targeted therapies currently exist [1]. Unlike other breast cancer subtypes, TNBC is unresponsive to hormones (oestrogen, progesterone), and HER-2 targeted therapies [2]. The currently available standard treatments for TNBC are chemotherapy, surgery and radiation [1].

Amino acid-targeted therapies represent a targeted approach for the treatment of TNBC that carries minimal toxicity; amino acid-targeted approaches are currently being tested that aim to circumvent off-target toxicities have been utilised to treat cancer or are currently being tested in clinical trials [3]. This strategy is a desirable option because cancer cells frequently alter their metabolic and nutrient acquisition pathways to overcome numerous tumour microenvironmental stresses that threaten their survival and ability to proliferate [4]. Arginine, a semi-essential amino acid, is involved in numerous metabolic pathways [5, 6] and arginine deprivation has been used to treat cancer in clinical trials [7, 8] like normal cells which can de novo synthesise arginine, frequent deregulation of argininosuccinate synthase 1 (ASS1) in TNBC makes these cancer cells highly dependent on the uptake of extracellular arginine [9]. This arginine-auxotrophic phenotype is commonly seen in the metabolic reprogramming of cancer cells [10, 11]. Both in vivo and in vitro studies in TNBC have shown that arginine shortage impairs mitochondrial function, induces endoplasmic reticulum (ER) stress response, and causes tumour cell death [9, 11]. While the effects of arginine shortage on mitochondrial function are fairly well understood [10, 12–15], the underlying molecular mechanisms controlling the ER stress response to acute arginine withdrawal remain to be elucidated [10, 12, 13].

Typically, tumour microenvironmental stresses, such as nutrient starvation, disrupt ER and protein homoeostasis and cells counter by triggering the unfolded protein response (UPR) [16, 17]. UPR, considered the canonical ER stress response, serves to (1) attenuate protein translation, (2) upregulate ER-resident chaperones, and (3) activate the ER-associated degradation (ERAD) pathway [18]. Generally, the UPR is viewed as a cytoprotective response; however, prolonged or unresolved ER stress can activate cell death [19, 20].

Binding immunoglobulin protein (BiP), also known as 78 kDa glucose-regulated protein (GRP-78), or heat shock 70 kDa protein 5 (HSPA5) [21], an ER stress sensor and a master regulator of ER homoeostasis is typically upregulated in response to cellular stresses [22]. Upregulation of BiP is typically a hallmark of canonical ER stress response/UPR [23, 24]. In addition to playing an essential role in the UPR, BiP, a resident ER chaperon protein [25, 26], also functions in various other non-ER-related signalling pathways [27, 28]. Upregulation of BiP is associated with cancer progression, tumour survival and proliferation, chemoresistance, angiogenesis, and metastasis, making BiP a metastatic marker [29]. Therefore, it is not surprising that ER stress and UPR signalling have also been shown to be dysregulated in multiple cancers [30]. Given that cancer cells adapt UPR to alleviate ER stress as a survival strategy, there is an impetus to therapeutically target the stress response to treat cancer [31]. Thus, it is important that the mechanisms by which BiP is regulated in cancer cells located in a nutrient-poor environment upon nutrient-starvation therapy be mechanistically understood [13, 32, 33].

There is an intimate link between protein synthesis (mRNA translation) and nutrient availability [34, 35]. Translation is a fundamental process that consumes the highest fraction of energy in highly proliferative cells, such as cancer cells [36]. Amino acids, such as arginine, are known to control the rate of protein synthesis by directly acting on mTORC1 [37–41]. Indeed, arginine shortage leads to the inactivation of mTORC1 signalling [37, 42]. In addition, recent studies have reported that arginine shortage also regulates global translation independent of mTORC1 signalling, and rather by ribosome pausing [43]. Ribosome pausing is a phenomenon that, until recently, was only observed in bacteria [44, 45]. It affects the elongation phase of translation and prolongs the time required to complete translation of a given mRNA resulting in decreased translation efficiency and defective protein folding [46]. In bacteria, this phenomenon was triggered by the shortage of single amino acids and was dependent on tRNA aminoacylation abundance [47]. In contrast, mammalian cells utilise a different mechanism [48]. In the case of arginine, two arginine codons (“pause-site” codons) that promote ribosome pausing are utilised as a mechanism by mammalian cells when arginine is limited [43]. Conceptually, ribosome pausing in mammalian cells impacts global translation levels, yet it is independent of tRNA abundance [43]. However, specific targets of arginine shortage-induced ribosome pausing remain to be identified. Therefore, we hypothesise that arginine shortage regulates BiP protein translation via ribosome pausing to exacerbate ER stress.

Previously, we have shown that arginine shortage induces ER stress in ASS1-deficient MDA-MB-231 cells [10]. Here, we investigated how arginine regulates BiP abundance during ER stress. We show that arginine shortage, unlike other ER stressors, inhibits BiP protein translation via ribosome pausing at the two known arginine pause-site codons, CGC and CGU. Using stable cells overexpressing a mutated BiP sequence free of arginine pause-site codons, we found that cells were able to resist arginine shortage-induced ER stress. Additionally, we identified BiP downregulation as a main feature of the non-canonical ER stress response to arginine shortage, which was also accompanied by an increase in ATF4 expression, a lack of ATF6 truncation and a BiP-suppressed XBP1s induction upon arginine shortage. Our findings highlight BiP as a protector of arginine shortage-induced growth inhibition. Collectively, this study is significant as it identifies BiP as a novel target of arginine shortage-induced ribosome pausing.

## Results

### Arginine shortage reduces BiP protein levels

**Fig. 1:**
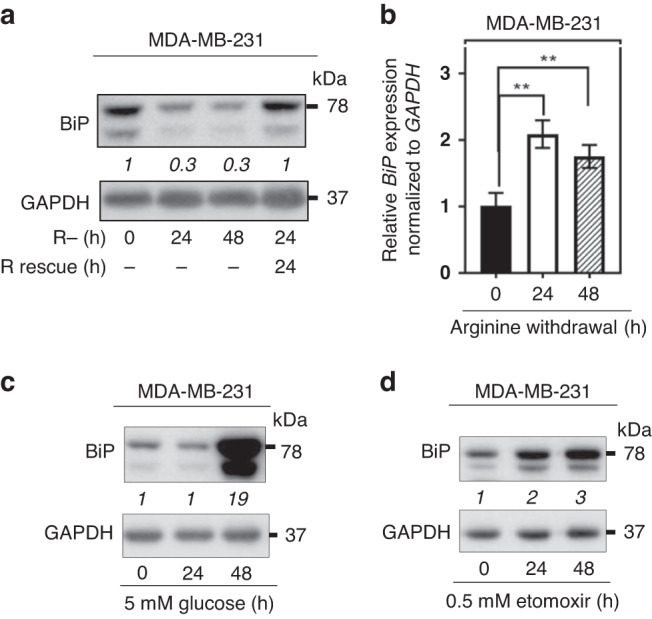
Arginine shortage uniquely affects BiP protein and mRNA levels in TNBC cells. **a** A representative Western blot shows BiP abundance in MDA-MB-231 cells grown either in arginine (control, 0 h) or arginine-free medium (R−) for the indicated time periods. Last lane*:* Arginine (84 mg/L, R rescue) was added to arginine-free medium for an additional 24 h. The relative BiP level (to the 0 h) is shown in italic after normalisation with the GAPDH level. **b** qRT-PCR analysis of *BiP* transcript levels in MDA-MB-231 cells under indicated arginine conditions. **c**, **d** A representative Western blot shows BiP abundance in MDA-MB-231 cells after treatment with glucose (5 mM, **c**) or etomoxir (0.5 mM, **d**) at the indicated time points. The densitometric analysis of a representative BiP as in **a**. Bars represent mean ± S.D.; *: *p* < 0.05; **: *p* < 0.01; (Student’s *t*-test); *n* = 3; R: arginine.

We hypothesised that *BiP* expression would be elevated in cancer cells based on prior work [29, 49]. Utilising data from The Cancer Genome Atlas (TCGA) Pan-Cancer project we compared *BiP* expression levels between primary tumours and normal tissues. We found that *BiP* message levels were significantly elevated in 12 of 17 cancer types, including breast cancer (Supplementary Fig. S1A). Next, we investigated *BiP* expression among the different breast cancer molecular subtypes and we found that basal-type TNBC showed the highest *BiP* expression levels compared to corresponding normal tissues, followed by the Her2 subtype (Supplementary Fig. S1B). A similar expression pattern was also observed in our analysis across 53 breast cancer cell lines from Cancer Cell Line Encyclopedia (CCLE; Supplementary Fig. S1C). Given that *BiP* expression was highest in TNBC, we next investigated whether BiP was involved in mediating arginine shortage-induced non-canonical ER stress in three TNBC cell lines and one non-TNBC cell line. We starved MDA-MB-231, BT-549, MDA-MB-468 and MCF-7 cells from extracellular arginine (R) for up to 48 h. After 24 or 48 h of arginine restriction, more than half of BiP protein abundance decreased in two of the four cell lines examined. Specifically, in MB-231 cells, there was a 0.7-fold decrease in BiP protein abundance, in BT-549 cells, there was a 0.2-fold decrease, and in MB-468 cells, there was a 0.8-fold decrease. (Fig. 1a, Supplementary Fig. S2A–C). Arginine replenishment for 24 h post-arginine removal restored BiP protein levels in MDA-MB-231 cells (Fig. 1a), indicating that it is a reversible process. Previous reports of BiP in different cellular compartments in response to ER stress [50] prompted us to examine and compare the changes of BiP in intracellular distribution patterns during arginine removal. The distribution change of BiP in MDA-MB-231 cells under arginine withdrawal was examined by cellular fractionation experiments. Cytoplasmic (without organelles) and nuclear fractions were prepared. We observed that the shortage of arginine for 24 and 48 h resulted in an increase in BiP protein abundance in the nucleus compared to the cytoplasm (Supplementary Fig. S3A). Interestingly, the abundance of nuclear BiP protein increased by twofold compared to the untreated control (Supplementary Fig. S3A), indicating the induction of ER stress. While our findings support the hypothesis that arginine restriction leads to ER stress, we also observed a decrease in total BiP protein abundance, which was not previously reported by others.

Next, to determine if the decrease in BiP protein abundance was unique to arginine shortage, we investigated the effects of different nutrient stressors on BiP abundance in MDA-MB-231 cells. Previous studies have shown that low glucose (5 mM) leads to the induction of BiP protein levels [51]. Therefore, we maintained MDA-MB-231 cells with glucose (5 mM) for up to 48 h, and we observed a dramatic increase in BiP protein abundance at 48 h post-treatment (Fig. 1c). The same trend was seen when the cells were treated with Etomoxir (0.5 mM), a fatty acid oxidation inhibitor. A twofold increase in BiP protein abundance after just 24 h of Etomoxir-treatment was observed compared to the control (Fig. 1d). Treatment of cells with lysine-free media resulted in an increase in both cytoplasmic and nuclear BiP protein levels (Supplementary Fig. S3B). After 24 and 48 h of lysine treatment, we observed a 0.05-fold and 0.27-fold increase in cytoplasmic BiP abundance, respectively, compared to the control. These results suggest that arginine shortage uniquely downregulates BiP protein abundance.

### Arginine shortage regulates BiP protein translation

**Fig. 2:**
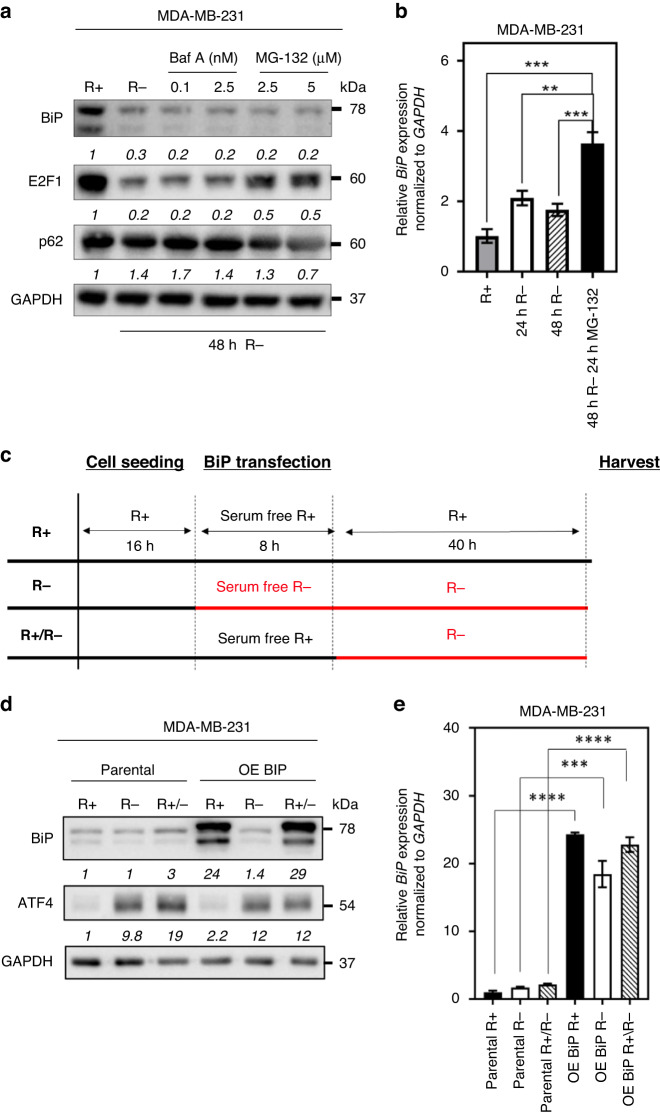
Arginine shortage inhibits BiP protein translation. **a** A representative image and densitometric analysis of Western blot of BiP, E2F1, and ubiquitin-binding protein p62 abundance in MDA-MB-231 cells grown under indicated conditions as in Fig. 1a (*n* = 3). **b** qRT-PCR analyses of *BiP* mRNA in MDA-MB-231 cells upon arginine starvation or arginine starvation in combination with MG-132 (*n* = 3). **c** Schematic showing workflow for (**d**, **e**). **d** Densitometric analysis of a representative Western blot of BiP and ATF4 abundance in parental MDA-MB-231 cells or cells transiently transfected with OE BiP and grown under conditions indicated in (**c**) (*n* = 3). **e** qRT-PCR analyses of *BiP* mRNA abundance in parental and OE BiP transfected MDA-MB-231 cells under conditions indicated in (**c**) (*n* = 3). Bars represent mean ± S.D.; *: *p* < 0.05; **: *p* < 0.01; ***: *p* < 0.001; ****: *p* < 0.0001 (Student’s *t*-test); R+: arginine present from transfection to harvest; R−: arginine removal from transfection to harvest; R+/R−: arginine removal from post-transfection to harvest; Baf A: Bafilomycin A1; R: arginine.

Protein expression can be regulated at several levels, including at the transcriptional level, translational level and also at the post-translational level [52, 53]. To achieve this goal, we first determined if arginine shortage downregulates *BiP* transcription. We measured *BiP* mRNA levels after treatment of MDA-MB-231 cells with arginine-free media for 24 and 48 h and found that arginine shortage withdrawal modestly increased *BiP* mRNA levels about twofold (Fig. 1b). It is likely that arginine regulates BiP protein abundance via a transcription-independent mechanism. Next, two common mechanisms that impact protein translation are the inhibition of protein synthesis and the acceleration of protein degradation [54]. To test if arginine shortage targets BiP for degradation, MDA-MB-231 cells deprived of arginine were exposed to either a lysosome inhibitor, Bafilomycin A, or a proteasome inhibitor, MG-132, for 48 h (Fig. 2a). We found that neither treatment was able to rescue BiP abundance, ruling out the involvement of autophagy or proteasome-mediated degradation was involved in regulating BiP abundance in the context of arginine shortage, where p62 was used as an internal control for Bafilomycin A treatment (Fig. 2a). Our internal control was E2F1, a transcription factor involved in regulating cell cycling [55]. E2F1 protein abundance, like Bip, also decreased upon arginine shortage alone. However, unlike BiP, E2F1 abundance was partially rescued upon treatment of MG-132 (0.3-fold to 0.5-fold respectively) (Fig. 2a). Interestingly, the combination treatment of MG-132 with arginine shortage (48 h) significantly increased *BiP* mRNA levels (Fig. 2b). Altogether, our results suggest that arginine withdrawal inhibits BiP protein abundance using a mechanism other than attenuating *BiP* transcription or accelerating its protein degradation.

To further assess how arginine affects BiP protein synthesis, we transiently overexpressed BiP in MDA-MB-231 cells under various arginine conditions (Fig. 2c). The control cells (R+) were never exposed to arginine shortage. The R− group, where arginine was present for cell seeding but was removed and changed to serum-free arginine-free media for the 8-h transfection and was then replaced with arginine-free media containing fetal bovine serum (FBS) for the remaining 40 h until harvesting. The last condition was the (R+/R−) group where arginine was present for cell seeding and the 8-h transfection but was switched to arginine-free media with FBS for the remaining 40 h (Fig. 2c). We found that removing arginine during transfection (R−) inhibited the overexpression of BiP protein (Fig. 2c). In contrast, BiP was overexpressed in R+/R− cells that were only deprived of arginine during the post-transfection period (Fig. 2d). We found that activating transcription factor 4 (ATF4), a marker of ER stress and UPR induction, was induced, supporting that the R− and R+/R− cells were indeed under nutrient stress conditions (Fig. 2d).

To further determine if the decrease of BiP protein abundance under the arginine-free condition (R−) did not result from transfection failure, we assessed *BiP* mRNA levels under all three experimental transfection conditions. We found that *BiP* mRNA was equally increased (by more than 15-fold) in all three conditions, showing the transfection was successful (Fig. 2e). Therefore, collectively our results suggest that arginine is required for *BiP* mRNA translation.

### Arginine shortage regulates BiP translation via ribosome pausing

Based on prior work [43], we hypothesised that arginine shortage leads to ribosome pausing and thereby inhibits BiP translation. To test this hypothesis, we first analysed all the arginine codons present in the *BiP* mRNA sequence. The arginine codons, CGC and CGU, were previously shown to be ribosome pause-sites [43]. We found that of the 28 arginine codons in the *BiP* sequence 11 of them (40%) were arginine pause-site codons (CGC and CGU) (Supplementary Fig. S4A). In contrast, when we assessed arginine codons in the *ATF4* sequence we found that less than 1% of the arginine codons were pause-site codons (Supplementary Fig. S4B). To determine if the pause-sites contribute to the inhibition of *BiP* mRNA translation upon arginine shortage, we converted all 11 arginine pause-site codons to an arginine non-pause-site codon (Fig. 4a). Next, we cloned the wild-type BiP sequence (WT BiP OE) and the mutated BiP sequence without arginine pause-sites (G-BiP OE), into a retroviral vector and then generated stable MDA-MB-231 cell lines overexpressing the two forms of *BiP* mRNA, respectively, After validation of transgene expression in full medium, we subjected parental MDA-MB-231 cells, WT BiP OE cells and mutated G-BiP OE cells to arginine shortage for up to 48 h (Fig. 4b). As expected, the steady-state level of BiP containing the arginine pause-site codons was sensitive to arginine shortage and still reduced in WT BiP OE cells (Fig. 4b). In contrast, the replacement of 11 arginine pause-site codons with non-pause-site codons rescued the steady-state BiP protein level and even increased at 24 h post-arginine shortage in G-BiP OE cells (Fig. 4b). However, at 48 h post-arginine shortage BiP expression was decreased in G-BiP OE cells, compared to the 24 h time point. Taken together, these observations indicate that indeed arginine shortage inhibits *BiP* translation via ribosome pausing after 24 h arginine shortage.

**Fig. 3:**
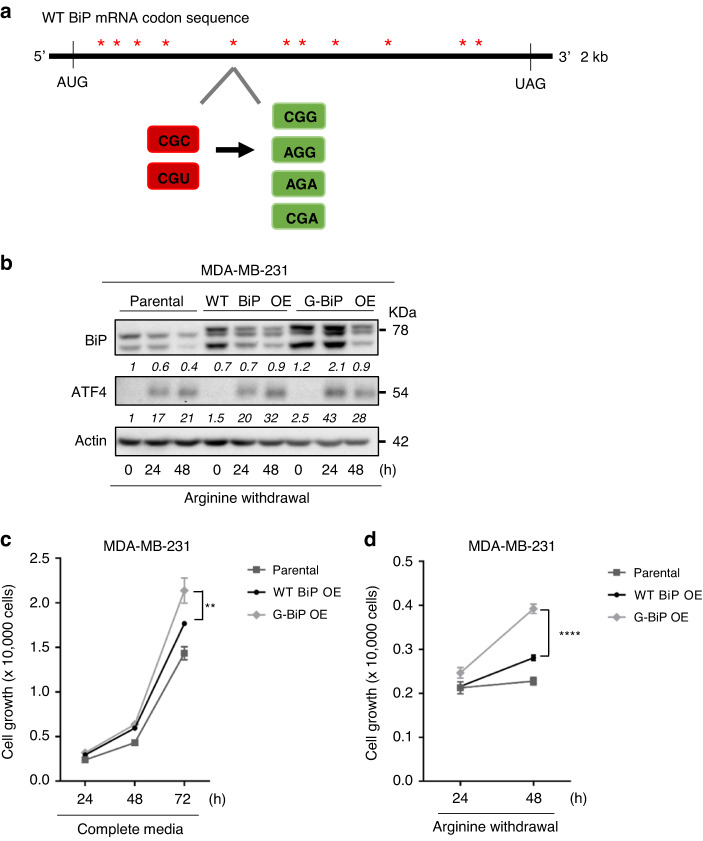
Arginine shortage regulates BiP translation via ribosome pausing. **a** Schematic illustrating the relative location of the 11 arginine pause-site codons (indicated as red _*_) in the *BiP* sequence that were mutated to non-pause-site codons to generate the G-BiP. **b** Densitometric analysis of a representative Western of BiP and ATF4 abundance in parental unmodified cells, and MDA-MB-231 cells with either WT BiP or G-BiP grown under indicated conditions as in Fig. 1a (*n* = 3). **c** Growth curve of parental MDA-MB-231 cells, WT BiP cells and G-BiP cells grown in full medium (*n* = 3). **d** Growth curve of parental MDA-MB-231 cells, WT BiP OE cells and G BiP cells in arginine-free medium (*n* = 3). Bars represent mean ± S.D.; *: *p* < 0.05; **: *p* < 0.01; ***: *p* < 0.001; ****: *p* < 0.0001 (Student’s *t*-test).

Next, we assessed the growth rate of WT BiP OE cells and G-BiP OE cells because overexpression of BiP was shown to increase cancer cell survival and tumorigenesis [56]. When arginine was available, we found that the G-BiP OE cells grew significantly faster compared to WT BiP OE cells and parental cells (Fig. 3c). Additionally, when arginine was removed, the G-BiP OE line exhibited a significant proliferative advantage over the WT BiP OE and parental lines (Fig. 3d). In summary, we found that ribosome pausing during arginine shortage directly leads to downregulation of BiP translation and that this impacts cell growth.

### Arginine shortage induces a non-canonical ER stress response in MDA-MB-231 cells

**Fig. 4:**
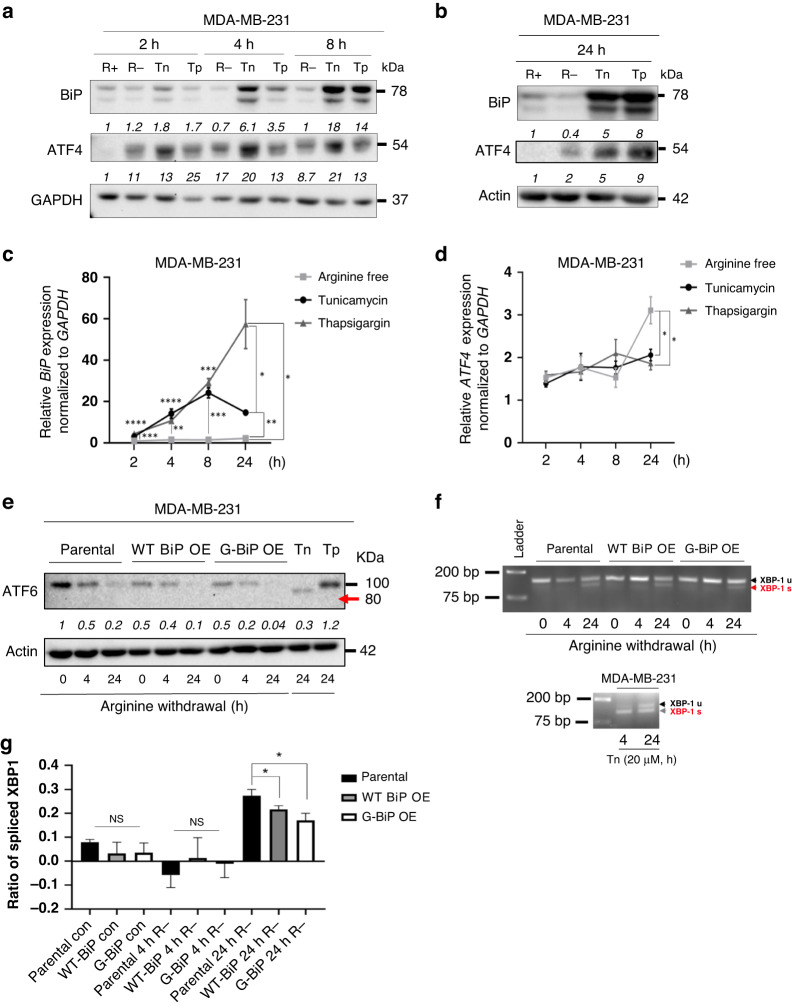
Arginine shortage induces non-canonical ER stress in MDA-MB-231 cells. **a**, **b** Densitometric analysis of a representative Western of BiP and ATF4 abundance in MDA-MB-231 cells subjected to indicated treatment conditions as in Fig. 1a (*n* = 3). **c**, **d** qRT-PCR analyses of *BiP* (**c**) and ATF4 (**d**) expression (*n* = 3). **e** Densitometric analysis of a representative Western of ATF6 abundance in MDA-MB-231 cells as in Fig. 1a (*n* = 3). **f** qRT-PCR analysis of *XBP1* mRNA alternative splicing in MDA-MB-231 cells harvested at indicated time points (*n* = 3). **g** Quantification of *XBP1* mRNA spliced ratio in cells treated under various conditions. Bars represent mean ± S.D.; *: *p* < 0.05 (Student’s *t*-test); Tn: Tunicamycin; Tg: Thapsigargin; R: arginine; XBP1u: inactive form of X box-binding protein 1; XBP1s: spliced-form of XBP1 which is its active form.

Next, we sought to understand the functional consequence of decreased BiP expression on the ER stress response, given that BiP is the master regulator of this process. ER stress triggers the activation of the UPR which is composed of three branches [57]. We first investigated the PERK branch by analysing its downstream effector, ATF4 during arginine shortage compared to other known ER stress inducers, Tunicamycin (Tn) and Thapsigargin (Tg) [58–60]. Tn is an antibiotic that prevents protein folding via inhibition of N-linked glycosylation, while Th is a sarco/endoplasmic reticulum Ca^2+^-ATPase (SERCA) inhibitor that causes Ca^2+^ depletion in the ER [60, 61]. Both inhibitors induce BiP expression and activate the UPR response [59, 62]. As expected, we found that Tn and Tg induced both BiP and ATF4 expression during various time points in MDA-MB-231 (Fig. 4a, b). In contrast, arginine shortage, compared to Tn and Tg, failed to induce BiP (Fig. 4a–c). Arginine shortage, Tn and Tg were all capable of inducing ATF4 protein and mRNA levels at all time points, serving as a control (Fig. 4b, d). This observation suggests that the upregulation of ATF4 expression is independent of BiP downregulation and that the canonical ER stress response is not induced upon arginine shortage in MDA-MB-231 cells.

Next, we assessed the ATF6 branch of the UPR [30]. Upon ER stress, ATF6 truncation is triggered to remove its cytosolic domain to allow it to enter the nucleus as a transcriptional factor [63]. To assess ATF6 activation, by arginine withdrawal, we investigated the status of ATF6 truncation under ± arginine conditions. We treated parental, WT BiP OE, and G-BiP OE lines with arginine-free media, Tn and Tg (Fig. 4e). Interestingly arginine shortage did not trigger ATF6 truncation, unlike Tn-treatment which resulted in the 80 kDa truncated form of ATF6 and Tg-treatment which increased the expression of full-length ATF6 at 100 kDa. This observation further suggests that arginine shortage induces a non-canonical ER stress response, in that it does not follow the canonical pathway of ATF6 truncation and activation.

**Fig. 5:**
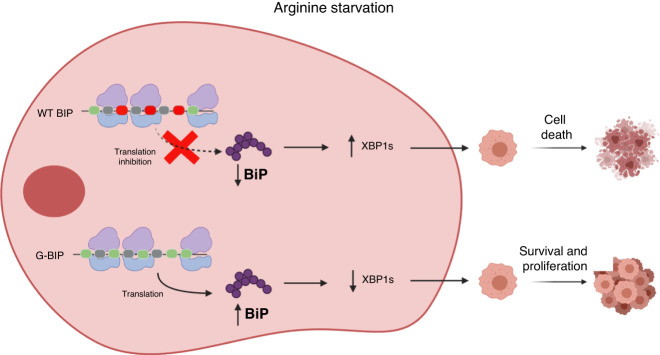
Proposed model outlining the effect of arginine shortage on BiP abundance and TNBC cell fate. Under arginine removal conditions, WT BiP is not able to be translated due to the presence of arginine pause-site codons. This leads to an increase in XBP1s and unresolved non-canonical ER stress and cell death in MDA-MB-231 cells. BiP translation is restored under arginine shortage conditions by removing the arginine pause-site codons. This allows the cell to survive arginine shortage-induced cell death, and the cells are able to continue to proliferate.

Lastly, we investigated the IRE1α branch of the UPR by assessing X box-binding protein 1 (XBP1) splicing. Upon UPR induction, IRE1α dimerises which triggers its ability to splice the mRNA encoding unspliced XBP1u to produce an active transcription factor, spliced XBP1 (XBP1s) [57]. XBP1s activates the transcription of genes involved in protein folding, including BiP [64–66]. Therefore, we analysed XBP1 RNA splicing in parental MDA-MB-231, WT BiP OE, and G-BiP OE cell lines under ± arginine conditions. We used qRT-PCR to assess for XBP1 splicing and plotted the ratio of spliced XBP1 under each condition. Arginine shortage was found to induce XBP1 splicing after 24 h irrespective of BiP status (Fig. 4f). However, quantitative analysis revealed that the parental MDA-MB-231 cells generated significantly more XBP1s compared to WT-BIP OE and G-BiP OE cell lines (Fig. 4g). There was an early induction of XBP1s in the parental line following Tn-treatment, compared to that in cells at 4 h post-arginine withdrawal (Fig. 4f). Interestingly, while Tn induced BiP at 4 and 24 h post-treatment, arginine shortage did not (Fig. 4a, b). Notably, the ratio of XBP1s is significantly decreased in WT BiP OE and G-BiP OE cells compared to parental MDA-MB-231 cells (Fig. 4g), this may contribute to their improved survival under arginine withdrawal condition (Fig. 3d and Fig. 5). Collectively, our results suggest that when arginine is removed, a non-canonical ER stress response is initiated that is characterised by the downregulation of BiP expression, the lack of ATF6 truncation and the decreased generation of XBP1s.

## Discussion

Although it has been previously shown that arginine is essential for protein translation via preventing ribosome pausing, specific protein targets of this mechanism had not been identified [43]. Herein, we report that arginine shortage selectively suppresses the translation of the master ER stress regulator, BiP in arginine-auxotrophic MDA-MB-231 cells. We determined this by removing all 11 arginine pause-sites, CGC and CGU, from the *BiP* mRNA sequence and replacing them with non-pause-site arginine codons. In doing so, we found that arginine shortage was no longer able to prevent BiP from being overexpressed in MDA-MB-231 cells. This mechanism has helped to explain the non-canonical ER stress response induced by arginine shortage. We found the non-canonical ER stress response to be characterised not only by the downregulation of BiP expression but also by the lack of ATF6 truncation and the decreased generation of XBP1s, compared to canonical ER stress. Significantly, our results highlight a novel mechanism by which arginine shortage is able to selectively suppress the translation of BiP and prevent cells from using canonical ER stress response for survival, thereby inhibiting cell growth.

In our study, we found that *BiP* expression was higher in most tumours, including TNBC, when compared to normal tissues (Supplementary Fig. S1). BiP is known to play a versatile role in cell survival due to that it antagonises apoptosis in response to ER stress and the UPR [57]. The canonical ER stress aims to restore ER homoeostasis and maintain cell viability and function by induction of the UPR [30]. Typically, BiP upregulation is a marker for canonical ER stress response [67]. Studies have highlighted that overexpression of BiP often induces BiP’s anti-apoptotic properties which include blocking the cleavage of procaspase-7 and -12, inhibiting stimulation of apoptotic proteins, such as BIK and BAX, and preventing cytochrome C release from the mitochondria [68]. Along the same line, many studies have reported that both high *BiP* mRNA and protein expression in cancer cells is correlated with cancer progression, drug resistance, and poor patient prognosis [27, 33, 69, 70]. For example, Roller et al. described BiP as a biomarker for chemoresistance due to numerous studies showing that high BiP expression correlates with resistance of breast cancer cells, glioblastoma, and leukaemia to chemotherapy [71–74]. Given that BiP is a key player for the canonical ER stress [23, 24], we were surprised to find that arginine shortage leads to a downregulation of BiP protein expression (Fig. 1a), even though *BiP* mRNA levels were upregulated (Fig. 1b). Interestingly, we found that while BiP protein levels were decreased in whole-cell extracts after arginine shortage when we isolated BiP protein from the cytoplasm and nucleus, we observed an increase in BiP abundance in the nucleus compared to the cytoplasm. This is consistent with previous findings [50].

A novel finding from our study is that arginine shortage regulates BiP protein translation. It has been established that amino acids regulate protein synthesis through various signalling pathways, including mTORC1 [37–41]. However, the unique contributions of specific amino acids to this process are not well understood. Indeed, there is evidence that arginine shortage negatively regulates protein translation by causing mRNA instability and that arginine shortage induces the phosphorylation of the eukaryotic initiation factor (elF) 2α [10, 75, 76]. These studies highlight the complex mechanisms involved in arginine’s regulation of translation. Consistent with this, we found that arginine shortage is able to selectively inhibit BiP translation by transiently expressing a BiP overexpression construct in MDA-MB-231 cells and showing that when cells were transfected in the absence of arginine, BiP was not detected (Fig. 2d). Interestingly we found that arginine withdrawal does not decrease global protein synthesis in MDA-MB-231 cells, given that ATF4 expression is significantly upregulated in response to arginine shortage (Fig. 2d). The selective inhibition of translation upon arginine shortage may be due to the presence and abundance of arginine pause-site codons. Previously, arginine limitation and not leucine limitation was shown to cause a decrease in translation via ribosome pausing [43]. Darnell et al. identified two arginine pause-site codons, CGC and CGU, at which they found ribosomes would stall, which led to elongation termination and inhibition of translation. Our study identifies BiP as a target for arginine shortage-induced ribosome pausing. Specifically, we found that under arginine shortage conditions, ribosome pausing indeed occurred at the two previously identified arginine pause-sites, CGC and CGU. This was made evident when we generated a mutant BiP sequence, G-BiP, that lacked CGC and CGU arginine codons. MDA-MB-231 G-BiP OE cells were able to overexpress BiP in the absence of arginine unlike WT BiP OE counterpart (Fig. 3b). This finding is significant as it identifies a specific target of ribosome pausing [43] in context of metabolic stress resulted from arginine shortage. We have previously demonstrated arginine shortage induces the expression of ER stress-responsive genes, such as *ATF4*, *ASNS* and *XBP*1 [10]. This activation of the UPR is further validated by an increase in markers like p-eIF2α, ATF4, and ASNS [10]. Consistent with our previous report, overexpression of BiP, regardless of WT or G-BiP, also induced ATF4 expression in response to arginine shortage (Fig. 3b).

Nitric oxide (NO) is produced by nitric oxide synthase (NOS) using the amino acid L-arginine as a substrate. Studies have shown that NO induces endoplasmic reticulum (ER) stress and increases the expression of BiP, a protein involved in ER stress response [77]. Additionally, NO also activates the UPR by promoting the splicing of the *XBP-1*, encoding a transcription factor, mRNA [78]. It is thought that NO may reduce ER stress by inducing UPR. NO also suppresses protein synthesis by increasing levels of p-eIF2α, a protein involved in regulating translation during stress conditions [79, 80]. It is possible that ribosome pausing represents an early response to extracellular arginine shortage, and additional mechanisms such as the complete depletion of intracellular arginine with prolonged arginine withdrawal, are responsible for the delayed downregulation of BiP levels.

Although we have identified BiP as a ribosome pausing target, it is still not clear as to what other proteins can be targeted via this mechanism. For example, ATF4 is consistently upregulated upon arginine shortage, independent of BiP abundance, whereas we found that ATF6 is downregulated, similar to BiP (Fig. 4). Interestingly, we found that 40% of the arginine codons present in the *BiP* mRNA coding sequence are pause-site codons (Supplementary Fig. S3A). Therefore, the abundance of arginine pause-site codons and their relative location in the mRNA may be a factor in determining arginine shortage-induced ribosome pausing. In addition, Darnell et al. used gene ontology to broadly assess enrichment related to the upregulation of arginine pause-sites and found that genes involved in nucleotide metabolism were less likely to contain arginine pause-sites, further highlighting the importance of arginine pause-site abundance [43]. Alternatively, it has been shown that tRNA abundance and synonymous codon usage influence translation in mammalian cells [81–83]. Many studies have shown that highly expressed genes are predominantly encoded by optimal codons therefore making their translation more efficient [79]. Interestingly in bacteria, limiting a single auxotrophic amino acid leads to the loss of tRNA charging and ribosome pausing at codons that are cognate to the limiting amino acid [84, 85]. The ribosome pausing that was observed in bacteria was shown to lead to abortive termination and an overall decrease in protein expression [44, 45, 47]. However, this phenomenon in mammalian cells has not been extensively studied in response to nutrient limitation. Further, studies have shown that when glutamine is limited, glutamine-specific tRNAs become selectively uncharged which contributes to decreased translation, however not due to ribosome pausing [86]. Overall, it would be interesting to determine if tRNA levels and tRNA charging influence the genes impacted by arginine shortage-induced ribosome pausing in TNBC cells.

Another significant finding from our study was that we found that preventing BiP downregulation allowed cells to proliferate in arginine shortage condition (Fig. 3d). One possible reason for the improved growth of the G-BiP OE cells in arginine shortage condition may because of the decreased XBP1 splicing ratio in the G-BiP OE cells [87] (Fig. 4g). The decrease in XBP1s ratio in G-BiP OE cells suggests that the XBP1 signalling pathway could be a potential target for TNBC therapies. Consistent with our finding (Fig. 3c), previous studies have shown that ribosome pausing is linked to reduced cell viability [43]. Additionally, Drummond and Wilke have suggested that ribosome pausing potentially has deleterious effects on the cell via protein misfolding- or mistranslation-induced cell stress [88], which is interesting as we show in arginine shortage-induced ER stress. Therefore, future studies are needed to determine if ribosome pausing is a targetable metabolic vulnerability, especially in cancer cell lines where the dysregulation of amino acid signalling is elevated [89, 90].

The limitations of this study are that we only look at this phenomenon in three TNBC cell lines. It would be interesting to determine if this mechanism occurs in other cancers such as prostate cancer and other breast cancer subtypes. Another limitation of our study is that we only perform in vitro studies, in future studies in vivo studies could help determine the effectiveness of BiP-inhibition as a therapy to treat TNBC.

## Materials and methods

### Cell lines and reagents

The human MDA-MB-231, BT-549, MDA-MB-468, MCF-7 and HEK293T cell lines were originally obtained from the American Type Culture Collection (ATCC). The cells were maintained in DMEM (Corning 10-013-CV) supplemented with 10% fetal bovine serum (FBS; Gibco, 10437028) at 37 °C in a humidified chamber at 5% CO_2_. Arginine-free DMEM (Thermo Scientific, 88364) was supplemented with 10% dialysed FBS (Gibco, 26400) and L-lysine (0.8 mM, Sigma-Aldrich, L5501). Lysine-free DMEM (Thermo Scientific, 88364) was supplemented with 10% dialysed FBS (Gibco, 26400) and L-Arginine (84 mg/L, Thermo Scientific A15738.22). MDA-MB-231 cells were initially cultured in regular DMEM until proper cell density was reached. Regular media was aspirated, and cells were washed briefly with PBS and continued to incubate in arginine-depleted DMEM for 4, 24, or 48 h at 37 °C or lysine-depleted DMEM for 24 or 48 h at 37 °C. Samples were harvested at each time point for either protein or RNA extraction. Etomoxir (Cayman Chemicals, 828934-41-4). Tunicamycin (T7765), Thapsigargin (T9033), Bafilomycin A1 (B1793), and MG-132 (M7449) were obtained from Sigma-Aldrich. MDA-MB-231 cells were pre-treated with arginine-free media for 24 h before the addition of either Bafilomycin A1 or MG-132 for 24 h. The cells were harvested for either protein or RNA after a combined 48 h of treatment.

### Preparation of nuclear and cytoplasmic extracts from MDA-MB-231 cells treated with arginine-free or lysine-free media

MDA-MB-231 cells were grown in 100-mm dishes and treated with either arginine-free media or lysine-free media once they reached 70% confluency. Both untreated controls and treated samples were harvested after either 24 or 48 h post-treatment. Nuclear and cytoplasmic extracts were prepared using NE-PER^TM^ Nuclear and Cytoplasm Extraction Reagents (Thermo Scientific, 78833) according to the manufacturer’s directions. In this protocol the cell membrane is disrupted using cytoplasmic extraction reagent I and cytoplasmic reagent II to release cytoplasmic contents, this is important in order to recover intact nuclei and to remove other membraned organelles. Cytoplasmic and nuclear extracts were stored in aliquots at −80 °C until used.

### Gene expression analysis

*HSAP5* gene expression data from the TCGA (The Cancer Genome Atlas) Pan-Cancer project [91] was used in this study. Log2-transformed RNA-Seq by Expectation-Maximisation (RSEM) expression values were used for boxplots. Statistical *p*-values between groups were determined by Wilcoxon tests. Also, the *HSPA5* expression in 53 invasive breast carcinomas from Cancer Cell Line Encyclopedia (CCLE) was downloaded from cBioPortal (http://www.cbioportal.org/) and compared. R version 4.0.5 was used for this analysis.

### Western blot and antibodies

Cells were harvested and lysed in 20 cell volumes of 1X SDS sample buffer and boiled for 10 min. The protein concentrations of whole-cell extracts were determined using a Bio-Rad Protein Assay Kit (500-0001; Bio-Rad). Protein samples (30–35 μg of total protein per lane) were loaded on gradient SDS-PAGE gels and transferred to PVDF membranes. The membranes were probed with antibodies specific to BiP, ATF4, E2F1, ATF6, (Cell Signaling Technology, 3177S, 11815S, 3742S, 65880 respectively), p62 (Proteintech, 18420-AP), GAPDH (Santa Cruz, sc-365645), and Actin (Millipore, MAB1501R). The membranes were incubated with primary antibodies overnight at 4 °C. After primary antibody incubation, blots were incubated with HRP-conjugated anti-rabbit or anti-mouse secondary antibodies (1:5000 dilution; Abcam, ab6721 and ab6789). Protein bands were visualised using chemiluminescence detection. Images were obtained and analysed using ChemiDoc Touch Imaging System and Image Lab software (Bio-Rad). To quantify the fold change of the desired protein bands, first, all bands were quantified by densitometry using Image Lab using the adjusted volume intensity setting. The intensity of the desired protein band for each sample was divided by GAPDH/Actin value with all samples from the same exposure, of course. This normalised BiP value was divided by the value for 0 h (=1) to determine the fold change; *n* = 3.

### RNA extraction and qRT-PCR analysis

Total RNA was extracted and purified using the Quick-RNA Miniprep Kit (Zymo, R1054). cDNA was synthesised using the iScript Kit (Bio-rad) and the qRT-PCR reaction utilised the components contained in the iTaq Universal SYBR Green Supermix (Bio-Rad, 1725120). The reaction was performed in the iQ5 Thermal Cycler (Bio-Rad) and data was analysed by the 2^−^^ΔΔCt^ method [92] and normalised to *GAPDH*; *n* = 3. The sequences of the primer pairs used are: *ATF4* (5’-CTCCAACATCCAATCTGTCCCG-3’ and 5’-TTCTCCAGCGACAAGGCTAAGG-3’), *BiP* (5’-CTGTCCAGGCTGGTGTGCTCT-3’ and CTTGGTAGGCACCACTGTGTTC-3’), *GAPDH* (5’-CCCCTTCATTGACCTCAACTA-3’ and 5’-CTCCTGGAAGATGGTGATGG), *XBP1* (5’-CCATGGGGAGATGTTCTGGAG-3’ and 5’-CCTGGTTGCTGAAGAGGAGG-3’).

### Cloning

G-BiP generation: Modified BiP sequence without arginine pause-sites was generated using ApE software. The sequence was uploaded to Integrated DNA Technologies to generate a Gblock DNA fragment. The Gblock DNA fragment was then inserted into pCMV BiP-Myc-KDEL-wt plasmid (Addgene 27164) and digested using HindIII and BamHI. We validated positive clones via Sanger sequencing carried out by the Integrative Genomics Core at City of Hope. Cloning into retroviral backbone: WT BiP sequence and G-BiP sequences were cloned into the pCMMP-BiP-IRES-mRFP plasmid (Addgene 36975) at Age1 and BamHI sites. Positive clones were verified via Sanger sequencing carried out by the Integrative Genomics Core at City of Hope. Sequencing primers: For G-BiP generation: GBiP-L 5’-CCAGAGGG CA GGAACACT-3’ and GBiP-R 5’-AGCAGGAGGAATTCCAGTCA-3’; for retroviral generation: pBABE 5’-CTTTATCCAGCCCTCAC-3’ and IRESreverse 5’-GCATTCCTTTGGCGAGAG-3’.

### Transfection

Transient transfection of pCMV BiP-Myc-KDEL-wt (1 µg) and pCMV BiP-Myc-KDEL-G-BiP (1 µg) was carried out using Lipofectamine 2000 (Life Technologies 11668-019) in MDA-MB-231 cells. Cells were harvested for Western blot analysis 40 h post-transfection. Cells were transfected under three conditions, the first was the R+ group, where arginine was present from cell seeding until the cells were harvested. The R− group had arginine present for cell seeding but was replaced with serum-free R− media for transfection and then replaced with R− media with FBS at 8 h post-transfection. The last group, R+/R−, had arginine present for cell seeding and transfection but was replaced by R− media with FBS until cells were harvested.

### Virus production and transduction

Target gene DNA in a retroviral backbone (pCMMP-BiP-IRES-mRFP) (1 µg), pCMV-VSV-G (250 ng), and pUMVC (750 ng) were transfected using Lipofectamine 2000 into HEK293T cells that had been seeded in a 6-well plate and reached 60% confluence on the day of transfection. The supernatant was harvested 24 and 48 h post-transfection and filtered through a 0.45-µm filter. For transduction, filtered supernatant containing viruses was added directly onto MDA-MB-231 cells and incubated overnight in the presence of polybrene (10 μg/mL). Forty-eight hours post-transduction, cells were passaged and screened for dsRED+ cells using flow cytometry. Sterile sort for dsRED+ cells was carried out to obtain a pure population of cells.

### Cell growth curve

The acid phosphatase (ACP) assay was used to measure cell growth. MDA-MB-231 cells, WT BiP OE cells and G-BiP OE cells were seeded at 7000 cells per well in 96-well plates. At the end of the indicated time periods, with and without arginine-free media treatment, cells were washed twice with PBS and then incubated at 37 °C for 30 min with 100 μL of pNPP (p-nitrophenol phosphate) solution (5 mM) in a buffer containing sodium acetate (0.1 M) and Triton X-100 (0.1% (v/v), pH 5.5). The reaction was terminated by adding NaOH (1 N, 10 μL), and the absorbance was measured at 410 nm using a microplate reader.

### Statistical analysis

Data with error bars are presented as a mean ± standard deviation. Student’s two-tailed *t*-test and Welch’s *t*-test were used to determine *p*-values, as indicated in figure legends. Differences were considered statistically significant when the *p*-value was <0.05.

## Conclusions

Collectively, our study identifies for the first time BiP as a specific target of arginine shortage-induced ribosome pausing. We show that BiP translation is inhibited upon arginine shortage due to ribosome pausing at two arginine pause-site codons. We also highlight the BiP’s protective role against arginine shortage-induced cell growth inhibition as a non-canonical ER stress. Overall, these findings are significant as they identify BiP as a novel target to inhibit TNBC cell growth and highlight ribosome pausing as a potential mechanism that can be utilised as an adjuvant after radio or chemotherapy to kill resistant TNBC cancer cells that survived the primary treatment, or they could use it as a neoadjuvant, to shrink the TNBC tumour before surgery.

## Supplementary information

Supplemental Figure Legend

Revised Supplemental Figure S1

Revised Supplemental Figure S2

Revised Supplemental Figure S3

Revised Supplemental Figure S4

Checklist

## Data Availability

N/A
