# Supplementary material for: Arginine regulates HSPA5/BiP translation through ribosome pausing in triple-negative breast cancer cells
Source: Br J Cancer. 2023 Jun 29;129(3):444–54. doi: 10.1038/s41416-023-02322-x (PMC10403569; doi:10.1038/s41416-023-02322-x)
Supplement: Supplementary file 1 — Supplemental Figure Legend [file 41416_2023_2322_MOESM1_ESM.pdf]

Supplemental Materials

Supplemental Figure S1. BiP-encoding gene *HSPA5* expression is elevated across human cancer types. (**A, B**) Standard boxplots are applied to visualize the log2-transformed BiP-encoding gene *HSPA5* expression levels (RSEM+1) between normal tissues (blue) and primary tumors (red) across 17 cancer types in TCGA Pan-Cancer study (**A**) and six breast cancer subtypes (**B**). Cancer types having more than five samples of normal tissues were included in this analysis. The number of samples are labeled at the bottom. Statistical p-values between groups were determined by Welch's t-tests; *: $p<0.05$; **: $p < 0.01$; ***: $p<0.001$; N/S: $p>0.05$. BLCA: bladder urothelial carcinoma; BRCA: breast invasive carcinoma; CHOL: cholangiocarcinoma; COAD: colon adenocarcinoma; ESCA: Esophageal carcinoma; HNSC: head and neck squamous cell carcinoma; KICH: kidney chromophobe; KIRC: kidney renal clear cell carcinoma; KIRP: kidney renal papillary cell carcinoma; LIHC: liver hepatocellular carcinoma; LUAD: lung adenocarcinoma; LUSC: lung squamous cell carcinoma; PRAD: prostate adenocarcinoma; READ: rectal adenocarcinoma; STAD: Stomach adenocarcinoma; THCA: Thyroid carcinoma; UCEC: uterine corpus endometrial carcinoma. (**C**) *HSPA5* expression data of 53 breast cancer cell lines from Cancer Cell Line Encyclopedia (CCLE), grouped by molecular subtypes, were compared. MDA-MB231 is labelled red.

Supplemental Figure S2. BiP expression upon arginine shortage. (**A, B, C**) A representative Western blot is shown (*left panel*). The quantification for BiP following densitometric tracing and normalization (*right panel*) in BT-549 cells (**A**) MDA-MB-468 cells (**B**) and MCF-7 cells (**C**) (n=3) grown either in full medium (control, 0 h) or arginine-free medium for the indicated time periods. Bars represent mean ± S.D.; **: $p < 0.01$; ***: $p<0.001$ (student's t test).

Supplemental Figure S3. The expression of BiP in cytoplasm and nucleus under conditions of arginine and lysine shortage was investigated. MDA-MB-231 cells were grown in either arginine-free medium (R-) (**A**) or lysine-free medium (**B**) for different time periods. A representative Western blot was used to determine the abundance of BiP in the cytoplasm and nucleus. Each lane contained 25 µg of protein. After normalization with GAPDH level, the relative cytoplasmic BiP levels are shown in italic, while the nuclear BiP levels were normalized to lamin B level. The results are presented below.

Supplemental Figure S4. Presence of Arginine pause-site and non-pause-site codons in *BiP* and *ATF4* mRNA. (**A, B**) Arginine pause-site codons in red and non-pause-site codons in green in the coding region of BiP (**A**) and ATF4 (**B**).
