# Supplementary figures and images for: Arginine regulates HSPA5/BiP translation through ribosome pausing in triple-negative breast cancer cells
Source: Br J Cancer. 2023 Jun 29;129(3):444–54. doi: 10.1038/s41416-023-02322-x (PMC10403569; doi:10.1038/s41416-023-02322-x)

A

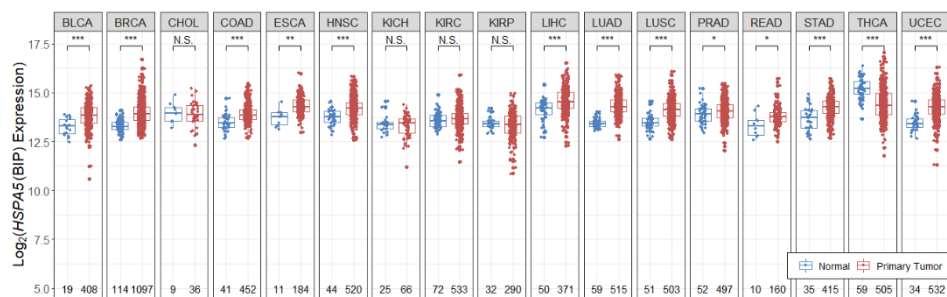

B

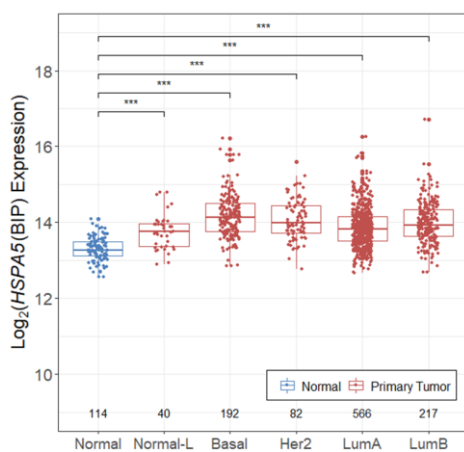

C

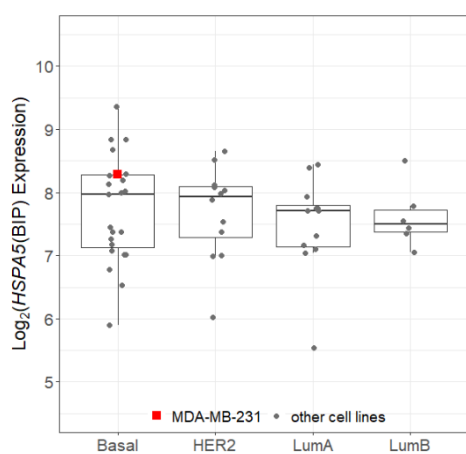

Supplement: Supplementary file 2 — Revised Supplemental Figure S1 [file 41416_2023_2322_MOESM2_ESM.pdf]

A

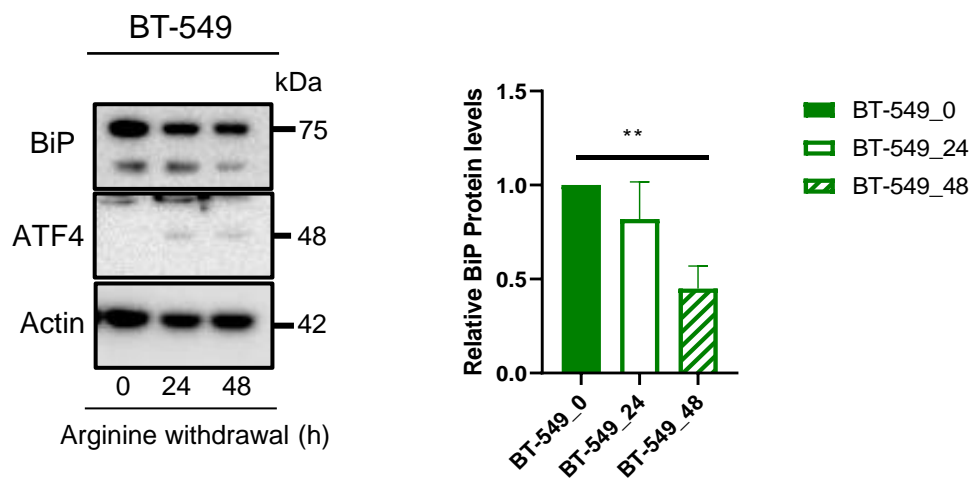

B

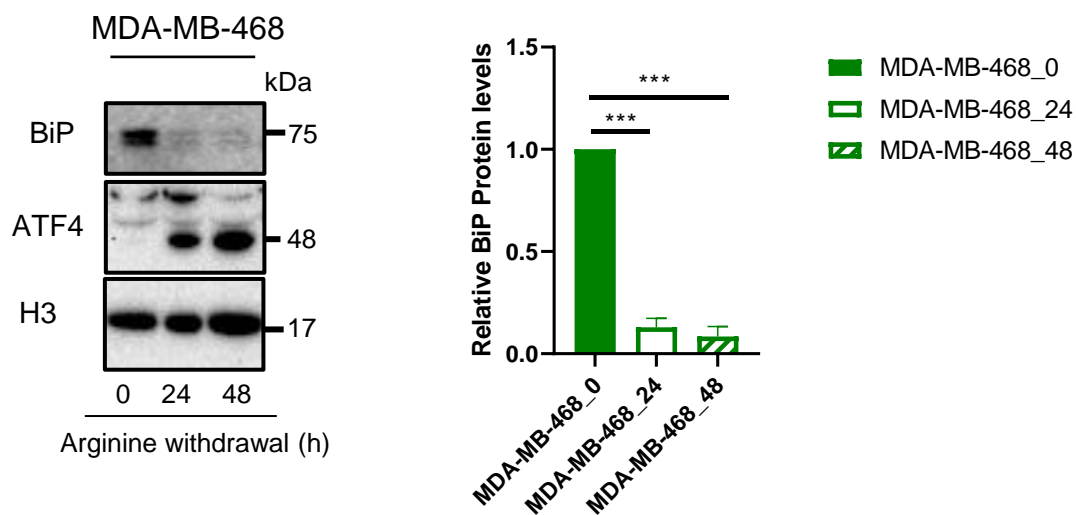

C

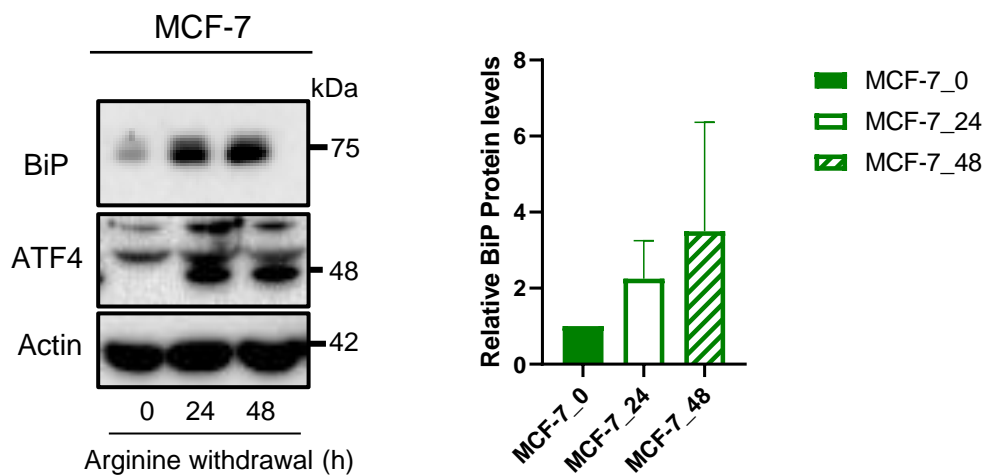

Supplement: Supplementary file 3 — Revised Supplemental Figure S2 [file 41416_2023_2322_MOESM3_ESM.pdf]

A

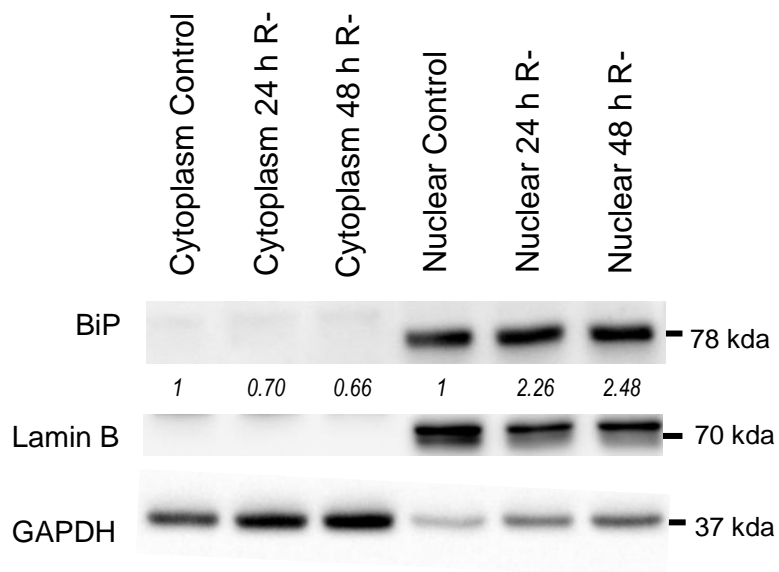

B

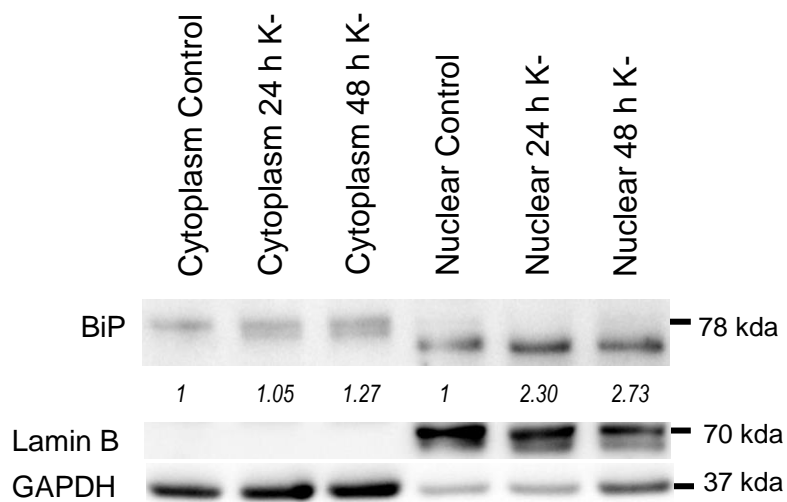

Supplement: Supplementary file 4 — Revised Supplemental Figure S3 [file 41416_2023_2322_MOESM4_ESM.pdf]
