# Supplementary material for: Arginine regulates HSPA5/BiP translation through ribosome pausing in triple-negative breast cancer cells
Source: Br J Cancer. 2023 Jun 29;129(3):444–54. doi: 10.1038/s41416-023-02322-x (PMC10403569; doi:10.1038/s41416-023-02322-x)
Supplement: Supplementary file 5 — Revised Supplemental Figure S4 [file 41416_2023_2322_MOESM5_ESM.pdf]

A

BiP (NM_005347.5) mRNA coding sequence

AUG	AAG	CUC	UCC	CUG	GUG	GCC	GCG	AUG	CUG	CUG	CUG	CUC	AGC	GCG	GCG	CGG	GCC	GAG	GAG	GAG	GAC	AAG	AAG	GAG
GAC	GUG	GGC	ACG	GUG	GUC	GGC	AUC	GAC	CUG	GGG	ACC	ACC	UAC	UCC	UGC	GUC	GGC	GUG	UUC	AAG	AAC	GGC	CGC	GUG
GAG	AUC	AUC	GCC	AAC	GAU	CAG	GGC	AAC	CGC	AUC	ACG	CCG	UCC	UAU	GUC	GCC	UUC	ACU	CCU	GAA	GGG	GAA	CGU	CUG
AUU	GGC	GAU	GCC	GCC	AAG	AAC	CAG	CUC	ACC	UCC	AAC	CCC	GAG	AAC	ACG	GUC	UUU	GAC	GCC	AAG	CGG	CUC	AUC	GGC
CGC	ACG	UGG	AAU	GAC	CCG	UCU	GUG	CAG	CAG	GAC	AUC	AAG	UUC	UUG	CCG	UUC	AAG	GUG	GUU	GAA	AAG	AAA	ACU	AAA
CCA	UAC	AUU	CAA	GUU	GAU	AUU	GGA	GGU	GGG	CAA	ACA	AAG	ACA	UUU	GCU	CCU	GAA	GAA	AUU	UCU	GCC	AUG	GUU	CUC
ACU	AAA	AUG	AAA	GAA	ACC	GCU	GAG	GCU	UAU	UUG	GGA	AAG	AAG	GUU	ACC	CAU	GCA	GUU	GUU	ACU	GUA	CCA	GCC	UAU
UUU	AAU	GAU	GCC	CAA	CGC	CAA	GCA	ACC	AAA	GAC	GCU	GGA	ACU	AUU	GCU	GGC	CUA	AAU	GUU	AUG	AGG	AUC	AUC	AAC
GAG	CCU	ACG	GCA	GCU	GCU	AUU	GCU	UAU	GGC	CUG	GAU	AAG	AGG	GAG	GGG	GAG	AAG	AAC	AUC	CUG	GUG	UUU	GAC	CUG
GGU	GGC	GGA	ACC	UUC	GAU	GUG	UCU	CUU	CUC	ACC	AUU	GAC	AAU	GGU	GUC	UUC	GAA	GUU	GUG	GCC	ACU	AAU	GGA	GAU
ACU	CAU	CUG	GGU	GGA	GAA	GAC	UUU	GAC	CAG	CGU	GUC	AUG	GAA	CAC	UUC	AUC	AAA	CUG	UAC	AAA	AAG	AAG	ACG	GGC
AAA	GAU	GUC	AGG	AAA	GAC	AAU	AGA	GCU	GUG	CAG	AAA	CUC	CGG	CGC	GAG	GUA	GAA	AAG	GCC	AAA	CGG	GCC	CUG	UCU
UCU	CAG	CAU	CAA	GCA	AGA	AUU	GAA	AUU	GAG	UCC	UUC	UAU	GAA	GGA	GAA	GAC	UUU	UCU	GAG	ACC	CUG	ACU	CGG	GCC
AAA	UUU	GAA	GAG	CUC	AAC	AUG	GAU	CUG	UUC	CGG	UCU	ACU	AUG	AAG	CCC	GUC	CAG	AAA	GUG	UUG	GAA	GAU	UCU	GAU
UUG	AAG	AAG	UCU	GAU	AUU	GAU	GAA	AUU	GUU	CUU	GUU	GGU	GGC	UCG	ACU	CGA	AUU	CCA	AAG	AUU	CAG	CAA	CUG	GUU
AAA	GAG	UUC	UUC	AAU	GGC	AAG	GAA	CCA	UCC	CGU	GGC	AUA	AAC	CCA	GAU	GAA	GCU	GUA	GCG	UAU	GGU	GCU	GCU	GUC
CAG	GCU	GGU	GUG	CUC	UCU	GGU	GAU	CAA	GAU	ACA	GGU	GAC	CUG	GUA	CUG	CUU	GAU	GUA	UGU	CCC	CUU	ACA	CUU	GGU
AUU	GAA	ACU	GUG	GGA	GGU	GUC	AUG	ACC	AAA	CUG	AUU	CCA	AGG	AAC	ACA	GUG	GUG	CCU	ACC	AAG	AAG	UCU	CAG	AUC
UUU	UCU	ACA	GCU	UCU	GAU	AAU	CAA	CCA	ACU	GUU	ACA	AUC	AAG	GUC	UAU	GAA	GGU	GAA	AGA	CCC	CUG	ACA	AAA	GAC
AAU	CAU	CUU	CUG	GGU	ACA	UUU	GAU	CUG	ACU	GGA	AUU	CCU	CCU	GCU	CCU	CGU	GGG	GUC	CCA	CAG	AUU	GAA	GUC	ACC
UUU	GAG	AUA	GAU	GUG	AAU	GGU	AUU	CUU	CGA	GUG	ACA	GCU	GAA	GAC	AAG	GGU	ACA	GGG	AAC	AAA	AAU	AAG	AUC	ACA
AUC	ACC	AAU	GAC	CAG	AAU	CGC	CUG	ACA	CCU	GAA	GAA	AUC	GAA	AGG	AUG	GUU	AAU	GAU	GCU	GAG	AAG	UUU	GCU	GAG
GAA	GAC	AAA	AAG	CUC	AAG	GAG	CGC	AUU	GAU	ACU	AGA	AAU	GAG	UUG	GAA	AGC	UAU	GCC	UAU	UCU	CUA	AAG	AAU	CAG
AUU	GGA	GAU	AAA	GAA	AAG	CUG	GGA	GGU	AAA	CUU	UCC	UCU	GAA	GAU	AAG	GAG	ACC	AUG	GAA	AAA	GCU	GUA	GAA	GAA
AAG	AUU	GAA	UGG	CUG	GAA	AGC	CAC	CAA	GAU	GCU	GAC	AUU	GAA	GAC	UUC	AAA	GCU	AAG	AAG	AAG	GAA	CUG	GAA	GAA
AUU	GUU	CAA	CCA	AUU	AUC	AGC	AAA	CUC	UAU	GGA	AGU	GCA	GGC	CCU	CCC	CCA	ACU	GGU	GAA	GAG	GAU	ACA	GCA	GAA
AAA	GAU	GAG	UUG	UAG																				

B

ATF4 (NM_001675.4) mRNA coding sequence

AUG	ACC	GAA	AUG	AGC	UUC	CUG	AGC	AGC	GAG	GUG	UUG	GUG	GGG	GAC	UUG	AUG	UCC	CCC	UUC	GAC	CAG	UCG	GGU	UUG
GGG	GCU	GAA	GAA	AGC	CUA	GGU	CUC	UUA	GAU	GAU	UAC	CUG	GAG	GUG	GCC	AAG	CAC	UUC	AAA	CCU	CAU	GGG	UUC	UCC
AGC	GAC	AAG	GCU	AAG	GCG	GGC	UCC	UCC	GAA	UGG	CUG	GCU	GUG	GAU	GGG	UUG	GUC	AGU	CCC	UCC	AAC	AAC	AGC	AAG
GAG	GAU	GCC	UUC	UCC	GGG	ACA	GAU	UGG	AUG	UUG	GAG	AAA	AUG	GAU	UUG	AAG	GAG	UUC	GAC	UUG	GAU	GCC	CUG	UUG
GGU	AUA	GAU	GAC	CUG	GAA	ACC	AUG	CCA	GAU	GAC	CUU	CUG	ACC	ACG	UUG	GAU	GAC	ACU	UGU	GAU	CUC	UUU	GCC	CCC
CUA	GUC	CAG	GAG	ACU	AAU	AAG	CAG	CCC	CCC	CAG	ACG	GUG	AAC	CCA	AUU	GGC	CAU	CUC	CCA	GAA	AGU	UUA	ACA	AAA
CCC	GAC	CAG	GUU	GCC	CCC	UUC	ACC	UUC	UUA	CAA	CCU	CUU	CCC	CUU	UCC	CCA	GGG	GUC	CUG	UCC	UCC	ACU	CCA	GAU
CAU	UCC	UUU	AGU	UUA	GAG	CUG	GGC	AGU	GAA	GUG	GAU	AUC	ACU	GAA	GGA	GAU	AGG	AAG	CCA	GAC	UAC	ACU	GCU	UAC
GUU	GCC	AUG	AUC	CCU	CAG	UGC	AUA	AAG	GAG	GAA	GAC	ACC	CCU	UCA	GAU	AAU	GAU	AGU	GGC	AUC	UGU	AUG	AGC	CCA
GAG	UCC	UAU	CUG	GGG	UCU	CCU	CAG	CAC	AGC	CCC	UCU	ACC	AGG	GGC	UCU	CCA	AAU	AGG	AGC	CUC	CCA	UCU	CCA	GGU
GUU	CUC	UGU	GGG	UCU	GCC	CGU	CCC	AAA	CCU	UAC	GAU	CCU	CCU	GGA	GAG	AAG	AUG	GUA	GCA	GCA	AAA	GUA	AAG	GGU
GAG	AAA	CUG	GAU	AAG	AAG	CUG	AAA	AAA	AUG	GAG	CAA	AAC	AAG	ACA	GCA	GCC	ACU	AGG	UAC	CGC	CAG	AAG	AAG	AGG
GCG	GAG	CAG	GAG	GCU	CUU	ACU	GGU	GAG	UGC	AAA	GAG	CUG	GAA	AAG	AAG	AAC	GAG	GCU	CUA	AAA	GAG	AGG	GCG	GAU
UCC	CUG	GCC	AAG	GAG	AUC	CAG	UAC	CUG	AAA	GAU	UUG	AUA	GAA	GAG	GUC	CGC	AAG	GCA	AGG	GGG	AAG	AAA	AGG	GUC
CCC	UAG																							

Supplemental Figure S4. Vidal *et al.* 2022
